# Editorial: Innovative drug combinations for enhanced solid tumor treatment efficacy

**DOI:** 10.3389/fonc.2025.1763808

**Published:** 2026-01-06

**Authors:** Giovanna Damia, Massimo Broggini

**Affiliations:** 1 Laboratory of Preclinical Gynaecological Oncology, Milan, Italy; 2 Laboratory of Molecular Pharmacology, Department of Experimental Oncology, Istituto di Ricerche Farmacologiche Mario Negri IRCCS, Milan, Italy

**Keywords:** chemotherapy, drug combination, drug resistance, immunotherapy, targeted therapy

Despite substantial advances in recent years, the treatment of solid tumors remains suboptimal. The identification of actionable molecular targets, the development of target-specific agents, and the advent of immunotherapy have not only profoundly reshaped the oncology landscape, but also clearly demonstrated that monotherapies are generally insufficient to elicit durable antitumor responses (1–5). The expanding understanding of the molecular mechanisms underlying tumor initiation, tumor progression and resistance provides an unprecedented foundation for the rational design of more effective treatment strategies, favoring and fostering the transition toward a personalized oncology medicine.

Importantly, this mechanistic knowledge is guiding the rational design of combination therapies, which represent the most promising approach to overcoming the intrinsic heterogeneity of solid tumors with the possibility to achieve more effective and potentially curative outcomes (6–9). Indeed, rationally designed combinations that integrate targeted agents, immunotherapies, or even conventional cytotoxic treatments have the potential to simultaneously suppress parallel oncogenic pathways, prevent compensatory signaling, and enhance antitumor immune responses (2, 7, 10, 11).

Efforts to develop, validate, and clinically implement well-designed combination regimens, based on the molecular characteristics of the tumors, are essential to achieve more effective, durable, and potentially curative outcomes for a broader population of patients. This was the goal of the Research Topic that collected 13 different contributions reporting different strategies converging on the use of new combinations.

In NSCLC the introduction of specific KRAS inhibitor has improved the outcome of patients harboring KRASG12C mutation. In their article, Tubita et al. showed that use of KRAS G12c inhibitors (both sotorasib and adagrasib) was able to enhance the response to conventional chemotherapy in NSCLC harboring this KRAS mutation. Using *in vitro* systems, the authors provide evidence that the superiority of the combination was obtained both using sequential and concurrent treatments and both in 2D and 3D models.

In the same setting (NSCLC), Jin et al. used a tri-specific antibody targeting EGFR, cMET and VEGF. The rationale behind the use of this tri-specific antibody is based on the known crosstalk between the three targeted signaling pathways. This strategy was superior to the use of single EGFR or bispecific EGFR/cMET antibodies. Furthermore, the authors showed that the strong efficacy of the trsipecific antibody could be even enhanced when combined with chemo or radiotherapy in xenografts models with strong and durable tumor regression without additional toxicity. Dual targeting of EGFR and VEGF has been tested clinically with a new third generation EGFR inhibitor (aumolertinib) in combination with an established anti-VEGF therapy (bevacizumab) in a phase II in patients with EGFR mutated NSCLC. The study (Kong et al.) reached the primary endpoint with an extension of progression free survival (PFS), again indicating the superiority of the simultaneously targeting pathways that are interconnected.

The use of antibodies targeting immune-checkpoint (PD1/PDL1 or CTLA4) has significantly changed the survival of NSCLC and melanoma patients. The combination of different checkpoint inhibitors represents now the standard treatment for these tumors. In a systematic review that included 10 clinical trials with more than 3000 patients, Dai et al. extended these results in gastrointestinal cancers, showing that the combination had strong efficacy in GI cancer and in particular in esophageal cancer again, reinforcing the superiority of combinations over monotherapy.

Two case reports, one in a patient with rare cervical sarcomatoid carcinoma (Zhang et al.) and the other in Grade 2 meningioma (Reusch et al.), highlighted the use of immunotherapy. In the first report, the authors used a combination of permbrolizumab (anti PD-1) with bevacizumab (targeting angiogenesis) that resulted in a prolonged PFS and OS. Interestingly, the combination was rationally designed based on the molecular characteristics of the patient, again supporting the notion that targeted combinations has stronger potential over empirical ones. The second case report indeed used immunotherapy as single therapy, based on the molecular characteristics of the tumor patient bearing a mutation in *PBMR1* (a gene involved in control of genomic stability) that, when mutated, in renal carcinoma associates with response to immune-checkpoint inhibitors.

Combinations of targeted therapy and chemotherapy have been tested in two case reports using as targeted agent anlotinib, a drug hitting several tyrosine kinases including VEGFR, FGFR, PDGFR and c-kit. In the first report (Sun et al.), the authors treated a rare lung NUT carcinoma (a very aggressive tumor with poor prognosis) with this multi-kinase inhibitor in combination with etoposide and cisplatin. The combination significantly prolonged PFS over that previously reported for this kind of tumor. The other case report (Liang et al.) was on a rare, poor prognosis, mesenchymal tumor (pulmonary arterial intima sarcoma) that was treated in a neo-adjuvant setting with the same multikinase inhibitor together with chemotherapy (in this case ifosfamide and pirarubicin). Interestingly, the patient was initially treated with chemotherapy alone, that resulted in a slow progression. Addition of anlotinib in subsequent treatments not only caused a significant tumor reduction, but also ameliorated the general symptoms of the patients.

Another important rational design for combinations includes the use of drugs targeting DNA repair. An interesting report (Zouggari et al.) showed that the addition of the thymidine analogue CldU was able to significantly enhance the response of *BRCA2*-mutated cells to olaparib (a PARP inhibitor). What is even more important, is that the use of CldU was able to overcome resistance to PARP inhibitors. This is particularly important because the efficacy of PARP inhibitors in clinic is hampered by the onset of drug resistance.

The review by Zhou et al. was centered on SLFN11 as a potential biomarker of sensitivity to DNA targeting agents. The authors gave an overview of the functions of SLFN11 but, in the context of the Research Topic, they proposed treatment strategies based on the expression status of SLFN11. This represents an additional way to foster combinations, as already discussed, based on the molecular characteristics of the tumors, and further prove that rational combination could even rise to synthetic lethality sparing normal cells.

Interestingly, in the manuscript by Chen et al. a screening of an in-house panel of small molecules against hepatocellular carcinoma (HCC) cancer stem cells led to the identification of a new compound C504244, able to interfere with the β -catenin signaling. Lenvatinib, a multi-targeted TKI, is approved for first-line treatments for advanced HCC. As resistance to lenvatinib has been associated to overactivation of β-catetin and that cancer stem cell have been implicated in therapy resistance in HHC, the authors combined C504244 and lenvatinib both *in vitro* and *in vivo* models and found how this combination reverted lenvatinib resistance.

A triple combination (palbociclib, a CDK4/6 inhibitor, PF-07104091, a CDK2 inhibitor and SX-682, a dual CXCR1 and CXCR2-CXCR1/2-) was tested as potential new effective treatment in preclinical models of melanoma (Yang et al.). The triple treatment was able not only to reduce melanoma tumor cell viability, to interfere with tumor growth more effectively in BRAF WT NRAS WT melanoma cells, but also induced a less immunosuppressive tumor immune microenvironment opening up the road for the design of clinical trials for immunochekpoint-resistant melanomas without BRAF mutation.

Finally, in the last manuscript (Zhang et al.), trastuzumab combined with low-dose nab-paclitaxel and radiotherapy was able to induce a very good disease control in a 86-years old man with a HER2-positive salivary duct carcinoma (SDC), a rare and aggressive malignancy. This case highlights the efficacy and safety of HER2-targeted combination therapy in elderly SDC patients, offering valuable insights into biomarker-driven personalized treatment strategies for this population.

All the manuscripts presented in this Research Topic support how rational, biology-guided combination strategies represent one of the most promising avenues to improve outcomes in solid tumors. Continued efforts to optimize and validate integrated therapeutic approaches will be crucial to translate these advances into durable and meaningful clinical benefits for patient with solid tumors.
